# 1069. Pharmacological preconditioning with vitamin c attenuates intestinal injury via the induction of heme oxygenase-1 after hemorrhagic shock in rats

**DOI:** 10.1186/2197-425X-2-S1-P85

**Published:** 2014-09-26

**Authors:** B Zhao, J Fei, Y Chen, X-Q Song, L Ma, L Wang, E-Z Chen, E-Q Mao

**Affiliations:** Department of Emergency Intensive Care Unit, Ruijin Hospital, Shanghai Jiaotong University School of Medicine, Shanghai, China

## Introduction

Pre-induction of heme oxygenase (HO)-1, which is regarded as an effective method of “organ preconditioning”, exerts beneficial effects during hemorrhagic shock (HS). However, the available HO-1 inducers exhibit disadvantages such as toxicity or complex technical requirements. Therefore, a safe and convenient HO-1 inducer would be promising and could be exploited in the treatment of foreseeable hemorrhaging, such as prior to major surgery. Recently, vitamin C (VitC) has been shown to attenuate organ injuries and inhibit inflammatory responses in hemorrhagic shock [3], but the specific mechanism remains unclear. Studies on the relationship between HO-1 and VitC are limited, and the results are controversial[4, 5].

## Objectives

We investigated the effect of vitamin C (VitC) on intestinal HO-1 expression and the involved mechanism. We further investigated if VitC pretreatment prevented HS related intestinal tissue injuries via HO-1 induction.

## Methods

The IEC-6 were treated with grade concentration of VitC as well as SB203580, PD98059 and SP600125, the inhibitors of p38 mitogen-activated protein kinase (MAPK), extracellular signal-regulated kinase (ERK) 1/2 and c-Jun N-terminal kinase (JNK). SD rats were pretreated with VitC (intraperitoneally, 100mg/Kg), HS was induced by drawing blood from the rat femoral artery (mean arterial pressure = 30 mm Hg) for 1 hr and resuscitating with the shed blood and Ringer's solution. Some rats further received zinc protoporphyrin (Znpp, intraperitoneally, 3 mg/kg), a HO-1 inhibitor.

## Results

The *in vitro* study showed HO-1 was induced in IEC-6 cell in a time- and concentration- dependent manner by VitC, and the inhibitor of ERK1/2 PD98059 inhibited the VitC induced HO-1 expression. The *in vivo* study showed the HO-1 protein (mainly observed in intestinal epithelial cells) and activity in intestine were highly induced in normal rat, and these HO-1 levels were further enhanced in HS rat model. The histological damage, apoptosis (number of TUNEL positive cell, Bcl-2/Bax ratio), neutophil infiltration (number of MPO positive cells, MPO activity and MPO protein level), and inflammatory cytokines level of tumor necrosis factor-a and interleukin-6 were all relieved by VitC pretreatment, and the protective effect of VitC was attenuated by Znpp.

**Figure 1:**
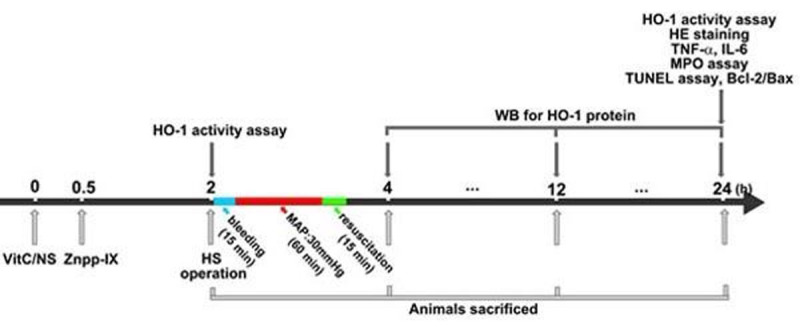
The schematic diagram of the main in vivo protocol

## Conclusions

These data suggests VitC might be applied as a safe inducer of intestinal HO-1 and VitC pretreatment attenuated HS related intestinal injuries via induction of HO-1 by activating ERK1/2 pathway.
